# Extended normobaric hyperoxia therapy yields greater neuroprotection for focal transient ischemia-reperfusion in rats

**DOI:** 10.1186/2045-9912-4-14

**Published:** 2014-08-10

**Authors:** Zhongrui Yuan, Rong Pan, Wenlan Liu, Ke Jian Liu

**Affiliations:** 1 College of Pharmacy, University of New Mexico Health Sciences Center, MSC09 5360, Albuquerque, NM 87131-0001, USA; 2 College of Medicine, Shandong University, Jinan 250012, China

**Keywords:** Apoptosis, Ischemic stroke, Normobaric hyperoxia, Oxidative stress, Tissue oxygenation

## Abstract

**Background:**

Normobaric hyperoxia (NBO) therapy is neuroprotective in acute ischemic stroke. However, how long the NBO should last to obtain optimal outcome is still unclear. Reports show that ischemic penumbra blood supply may remain compromised for a long period after ischemia-reperfusion, which would impair tissue oxygenation in ischemic penumbra. Therefore, we hypothesized that longer-lasting NBO may yield greater neuroprotection.

**Methods:**

The relationship between treatment outcome and NBO duration was examined in this study. Rats were subjected to 90 min middle cerebral artery occlusion followed by reperfusion for 22.5 hours. NBO started at 30 min post ischemia and lasted for 2, 4 or 8 h. Treatment efficacy was evaluated by measuring infarction volume, oxidative stress and apoptosis.

**Results:**

Among 2 h, 4 h and 8 h NBO, 8 h NBO offered the greatest efficacy in reducing 24-hour infarction volume, attenuating oxidative stress that was indicated by decreased production of 8-hydroxydeoxyguanosine and NADPH oxidase catalytic subunit gp91^phox^, and alleviating apoptosis that was associated with reduced production of DNA fragment and caspase-3 activity in cortex penumbra.

**Conclusions:**

Under our experimental conditions, longer duration of NBO treatment produced greater benefits in focal transient cerebral ischemia-reperfusion rats.

## Introduction

Accumulating evidence demonstrates that normobaric hyperoxia (NBO) therapy could be a clinically viable adjunct method for thrombolysis in acute ischemic stroke. Several animal [1-5] and human [6-8] studies have documented that NBO is neuroprotective in acute ischemic stroke, improving pathologic, neurobehavioral and neuroimaging outcomes, without increasing markers of oxidative stress. Moreover, several recent animal studies [5,9] have shown that NBO increases the safety and efficacy of thrombolysis in acute ischemic stroke. In addition, NBO is simple to administer, noninvasive, inexpensive, widely available, and can be started in the field within a relatively short time after stroke symptom onset, although NBO may not be as efficient as hyperbaric oxygen therapy [10].

The optimal outcome of NBO therapy may depend on various parameters and conditions. Singhal and his colleagues have documented that initiating treatment at earlier time points after stroke onset enhances the degree of neuroprotection [11]. Beneficial outcome was observed with NBO at any stage of experimental stroke, and maximum benefit was evident with continuous (both intra-ischemic and post-ischemic) treatment [1]. But how long the NBO should last to obtain the most desirable outcome is still unclear. We have reported that up to 15% of the capillaries in the ischemic penumbra remained occluded at least 2 hours after reperfusion following 90 min cerebral ischemia [12]. Moreover, on a long-term basis, heart tissue perfusion after ischemia-reperfusion remains markedly compromised for at least 4 weeks [13]. These findings suggest that ischemic penumbra blood supply may remain compromised for a long period after ischemia-reperfusion, which could significantly impair ischemic penumbra oxygen supply. Therefore, we hypothesized that longer-lasting NBO may yield greater neuroprotection. In this study, we investigated the efficacy of NBO with different duration for focal transient cerebral ischemia in rats by measuring the infarction volume, oxidative stress and apoptosis.

## Materials and methods

### Animal preparation

The Laboratory Animal Care and Use Committee of the UNM HSC approved all experimental protocols. Fifty three adult male Sprague–Dawley rats (280–320 g; Charles River Laboratories, MA) were used for all experiments. The animals were maintained in a climate-controlled vivarium with a 12 h light–dark cycle with free access to food and water.

For all surgical procedures, 4.0% isoflurane in 70% N_2_:30% O_2_ was used for anesthesia induction, and 1.75% for anesthesia maintenance. Physiologic monitoring during the procedure comprised measurement and maintenance of core (rectal) temperature at 37.5 ± 0.5°C using a heating pad.

Focal cerebral ischemia was produced by intraluminal suture occlusion of the right middle cerebral artery (MCA) using a 4–0 silicone-coated nylon filament as described previously [14]. The MCA was occluded for 90 min and reperfusion was produced by gently withdrawing the suture out of external carotid artery (ECA). For all animals used in this study, successful middle cerebral artery occlusion (MCAO) was confirmed in vivo by laser Doppler flowmetry (LDF) as described previously [15].

### Study design

**Figure 1:**
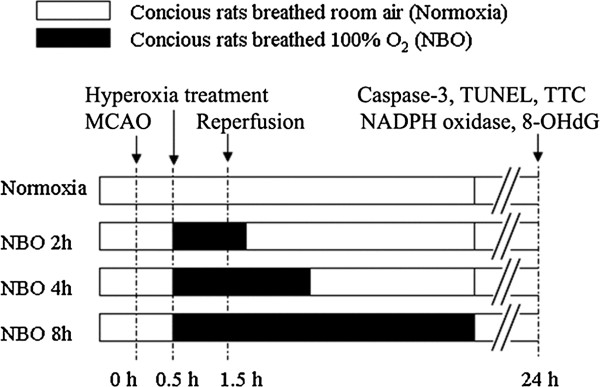
Schematic representation of the protocol illustrates the timing of middle cerebral artery occlusion (MCAO) and reperfusion, normobaric hyperoxia therapy (NBO), 2,3,7-triphenyltetrazolium chloride (TTC) staining, NADPH oxidase expression, 8-hydroxy-deoxyguanosine (8-OHdG) immunohistochemistry, caspase-3 activity, terminal deoxynucleotidyl transferase-mediated uridine 5’-triphosphate-biotin nick end labeling (TUNEL) staining.

This study was designed to investigate the neuroprotective potential of NBO when administrated for different durations (2, 4 or 8 h). Rats breathing room air were used as control. In the NBO groups, as shown in Figure 1, rats breathed 100% O_2_ at 30 min after ischemia in a chamber at a flow rate of 3.5 L/min and lasted for 2 h, 4 h and 8 h, respectively. The animals breathed spontaneously, and were not anesthetized except during surgery. The rats were sacrificed at 24 h after ischemia, the brains were quickly removed, and samples were collected for evaluation of infarction volume, oxidative stress and apoptosis.

### NBO treatment protocol

Thirty min after the onset of MCAO, anesthesia was discontinued, and the rats were put into an air-tight box (the same box used for anesthesia induction), which was ventilated (3.5 L/min) with 100% O_2_ or medical air (21% O_2_, air) until the end of 90-min MCAO. After MCAO, rats were anesthetized with isoflurane (4% for induction, 1.75% for maintenance) in N_2_O:O_2_ (70:30) during reperfusion procedures (10 min in total). Then, rats were put back into the air-tight box which was ventilated with 100% O_2_ or medical air to finish NBO or air treatment.

### Quantification of brain infarction

Rats were divided into four groups: Normoxia, NBO 2 h, NBO 4 h and NBO 8 h groups. There were six animals in each group quantifying brain infarction. Rats were decapitated at 24 hours after MCAO according to experimental design. Brains were removed and chilled with ice water for 5 min and sectioned into 2 mm thick slices using brain matrix and stained by immersion into 2% 2,3,5-triphenyltetrazolium chloride (TTC) stain as previously described [14]. Digital pictures were taken and the total (hemispheric), cortical, and subcortical (striatal) infarction volumes were calculated using Image Pro Plus (version 4.1) software.

### Brain tissue preparation

Twenty five Rats were divided into five groups: Sham, Normoxia, NBO 2 h, NBO 4 h and NBO 8 h groups. For sham group, the rats were subjected to the same surgical procedures, except the suture was advanced only 10 mm (not 19 mm) above the bifurcation and withdrawn after 10 seconds. At 24 h after ischemia, the animals were anesthetized with 3% isoflurane and perfused transcardially with heparined saline. The brains were removed quickly. Each brain was cut into eight 2-mm-thick coronal blocks using a rat brain matrix from the anterior surface to posterior. The third of the coronal blocks from each brain were fixed for four hours in 4% paraformaldehyde in 0.1 mol/L phosphate buffer, pH 7.4 and then was cryoprotected with a series of sucrose solutions (20%, 25%, and 30%) at 4°C, embedded in molds with embedding compound (Cryo OCT compound, Sakura Fineteck USA Inc, Torrance, CA), and stored at -70°C until sectioned. Consecutive coronal sections (10 μm), 1 mm from the anterior surface of each block, were cut on a cryostat. The sections were used for the following detection of oxidative DNA damage and DNA fragment by immunohistochemistry and TUNEL, respectively.

The cortex penumbra and corresponding contralateral tissue were sampled from the fourth and fifth of the coronal blocks from each brain as previously described [14]. Briefly, TTC staining was used to determine the infarction areas on the sliced brain tissue. Four rats underwent 24-hour permanent MCAO was used to demonstrate the maximum ischemically compromised area after MCAO. The difference of infarction area in the cortex between NBO treated group and permanent MCAO group was considered as the cortex penumbra. The samples were stored at -70°C until used. The frozen tissues were homogenized with a lysis buffer (Tris-Cl: 50 mmol/L pH 7.6, NaCl: 150 mmol/L, CaCl_2_: 5 mmol/L, Brij-35: 0.05%, NaN_3_: 0.02%, Triton X-100: 1%). Subsequently, after centrifugation, the supernatants from each sample were detected for the protein concentrations and the analysis of NADPH oxidase and caspase-3. The protein concentrations in the homogenates were determined using Bradford reagent (BioRad Laboratories, Hercules, CA, USA).

### NADPH oxidase detection by western blotting

To evaluate oxidative stress, we performed western blotting and immunohistochemistry to detect the expression of NADPH oxidase subunit gp91^phox^ and 8-hydroxy-deoxyguanosine (8-OHdG) to detect oxidative DNA damage, respectively.

Proteins (30 μg) from above extracted samples were electrophoresed in 10% SDS-PAGE acrylamide gels, transferred onto nitrocellulose membranes (BioRad Laboratories, Hercules, CA, USA), and incubated for 1 h in TBS containing 5% nonfat milk and 0.1% Tween-20 at room temperature. Membranes were then incubated overnight with polyclonal primary antibody against gp91^phox^ (1:1,000, BD Transduction Laboratories, USA), then washed in TBS with 0.1% Tween-20, incubated for 1 h at room temperature with HRP-conjugated anti-rabbit antibody (1:1,000, Santa Cruz Biotechnology Inc., USA), and revealed using the SuperSignal West Pico chemiluminescent kit (Pierce Biotechnology, Rockford, IL, USA) following the manufacturer’s instructions. To control sample loading and protein transfer, the membranes were stripped and rehybridized to assess *β*-actin (1:1,000, Santa Cruz Biotechnology Inc., USA). The gp91^phox^ was standardized in proportion to *β*-actin.

### Immunohistochemistry for 8-OHdG

Immunohistochemistry was performed on 10 *μ*m-thick sections which were prepared as described in *brain tissue preparation.* After incubation in 3% H_2_O_2_ followed by 10% normal goat serum in PBS Tween-20, the sections were strained overnight at 4°C using a mouse monoclonal antibody (1:1000, TREVIGEN, INC. MD, USA) against 8-OHdG to detect oxidative DNA damage. Sections were then treated with secondary antibody (BA-1000; Vector Laboratories, CA, USA). Immunoreactivity was visualized subsequently by the avidin-biotin complex method (Vector Laboratories, CA, USA) as described previously [16]. As a negative control, sections were incubated without the primary antibody.

The slides were mounted using mounting medium. Only strong staining was considered as 8-OHdG–positive and 8-OHdG-positive cells were quantified with a light microscope (Olympus BX-52). 8-OHdG-positive cells in the cortex penumbra region were counted under × 400 magnification in five to seven sections each animal. Results were expressed as the average number of cells per mm^2^ in the areas.

### In situ labeling of DNA fragmentation

To explore the possible mechanism of neuroprotection by NBO, DNA fragmentation and caspase-3 activity were assessed to evaluate the impact of NBO on apoptosis. Terminal deoxynucleotidyl transferase-mediated dUTP-biotin nick end labeling (TUNEL)-positive cells were detected by using NeuroTACS™ II *In Situ* Apoptosis Detection Kit (Trevigen, Inc., MD, USA). In brief, after endogenous peroxidase was inactivated with 3% H_2_O_2_ for 5 min, the sections were immersed in terminal deoxynucleotidyl transferase (TdT) buffer and incubated with Labeling Reaction Mix (TdT dNTP Mix, TdT Enzyme, TdT Labeling Buffer and Mn^2+^ Cation). The sections were incubated with avidin-biotin-horseradish peroxidase and visualized with 3 mmol/L 3,3’-diaminobenzidine tetrahydrochloride and 18 mmol/L hydrogen peroxide in PBS. As a negative control, sections were incubated without TdT Enzyme in the Labeling Reaction Mix. The slides were mounted using mounting medium. Only strong staining was considered as TUNEL–positive and TUNEL-positive cells were quantified with a light microscope (Olympus BX-52). TUNEL-positive cells in the cortex penumbra region were counted under × 400 magnification in five to seven sections each animal. Results were expressed as the average number of cells per mm^2^ in the areas.

### Caspase-3 assay

Caspase-3 activity in cortex penumbra was determined following the manufacturer’s instructions using an EnzChek® caspase-3 assay kit (Molecular Probes, Inc. USA). Briefly, supernatants, which were prepared as described in *brain tissue preparation,* from each sample were mixed with Z-Asp-Glu-Val-Asp-AMC substrate solution. A standard curve of AMC ranging from 0 to 100 *μ*mol/L was run with each set of samples. Negative control without enzyme was used in each assay to determine the background fluorescence of the substrate. As an additional control to some selected samples, 1 *μ*M of the 1 mM Ac-Asp-Glu-Val-Asp-CHO (aldehyde), the caspase-3 inhibitor stock solution, was added. Fluorescence was measured at an excitation wavelength of 342 nm and an emission wavelength of 441 nm in a Fluostar Optima fluorescence microplate reader (BMG LABTECH).

### Statistics

All data were expressed as mean ± SD. Differences between multiple groups were tested by ANOVA. Tukey–Kramer test was used as a post-hoc test. *P* values of less than 0.05 were considered as significant differences.

## Results

### The impact of NBO on infarct size

**Figure 2:**
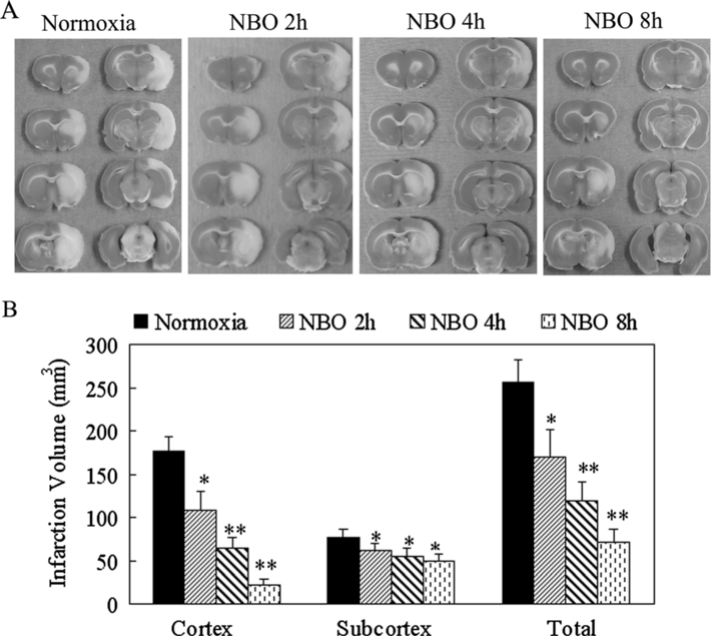
**The effect of NBO on the infarct volume.** The rats were sacrificed at 24 h after ischemia and TTC staining was performed. **A)** Representative photograph of TTC staining in each animal group. **B)** Histogram represents infarct volume. Data is expressed as mean ± SD, *n* = 6 in each group. *P < 0.05 and **P < 0.01 *vs.* normoxic group.

Infarct volume was assessed by TTC staining at 24 h after ischemia. As shown in Figure 2, the infarct volumes were (177 ± 15) mm^3^ (cortex), (77 ± 10) mm^3^ (subcortex) and (255 ± 25) mm^3^ (total) in normoxic control group. Compared with normoxic control, NBO significantly decreased total (hemispheric) infarct volume to (170 ± 30) mm^3^ (NBO 2 h), (119 ± 21) mm^3^ (NBO 4 h) and (71 ± 15) mm^3^ (NBO 8 h), especially cortical infarct volume to (109 ± 21) mm^3^ (NBO 2 h), (64 ± 12) mm^3^ (NBO 4 h), (22 ± 8) mm^3^ (NBO 8 h). NBO treatment remarkably decreased cortex infarct volume while there was no significant change on subcortex infarct volume. These results show that NBO is neuroprotective, and longer NBO duration produces better protection, particularly in the cortical region.

### Effect of NBO on NADPH oxidase

**Figure 3:**
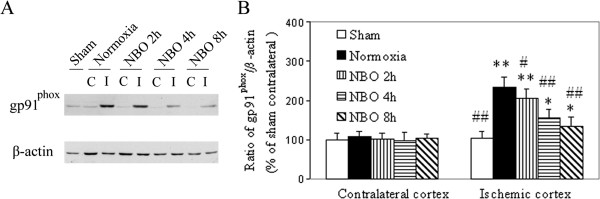
**The effect of NBO on NADPH oxidase subunit gp91**^**phox **^**in the cortex penumbra at 22.5 h reperfusion after 90 min ischemia. A)** Representative western blotting shows the expression of gp91^phox^. **B)** Histogram represents the relative amount of NADPH oxidase subunit gp91^phox^ normalized to the level of sham-operation contralateral. Values are expressed as mean ± SD, *n* = 5 each group. *P < 0.05 and **P < 0.01 *vs.* sham; # *P* < 0.05 and ## *P* < 0.01 *vs.* normoxia group.

NADPH oxidase is known to produce reactive oxygen species after cerebral ischemia and reperfusion [17]. Therefore, to determine the impact of NBO treatment on oxidative stress in cortex penumbra, we performed western blotting to detect gp91^phox^, a catalytic subunit of NADPH oxidase. We found that focal cerebral ischemia induced a substantial increase in the levels of gp91^phox^ in the cortex penumbra of normoxic rats (Figure 3). NBO markedly attenuated the up-expression of gp91^phox^ caused by ischemia in a NBO duration-dependent manner, decreasing the expression of gp91^phox^ in the cortex penumbra by 13%, 43% and 50%, respectively, with 2 h, 4 h or 8 h duration. These results suggest that NBO protect brain likely through the reduction of ischemia-induced oxidative stress.

### Oxidative DNA damage

**Figure 4:**
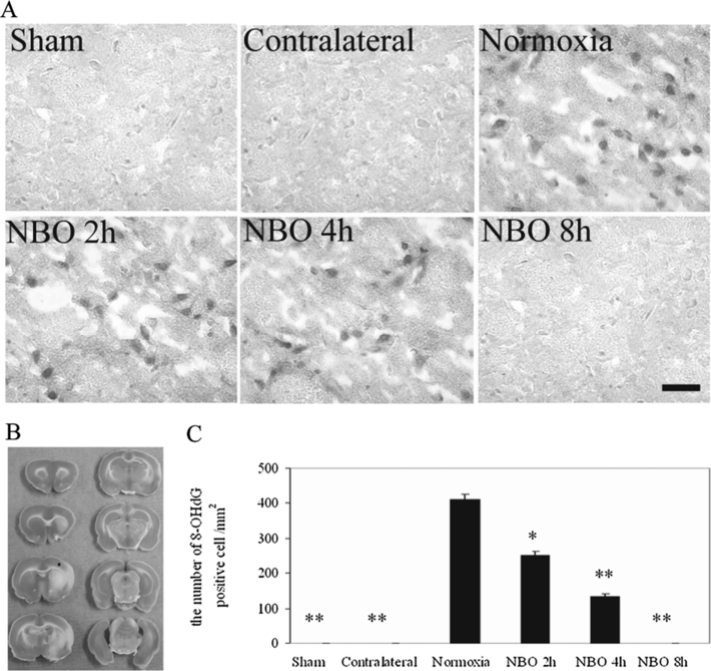
**The effect of NBO on the production of 8-OHdG in cortex penumbra at 22.5 h reperfusion after 90-min ischemia. A)** Representative photographs show the effect of NBO on the production of 8-OHdG in cortex penumbra. Bar = 20 *μ*m. **B)** TTC staining. The black square indicates the location where the photograph taken from. **C)** Numbers of 8-OHdG-positive cell in the cortex penumbra. Values are expressed as mean ± SD, *n* = 5 each group. *P < 0.05 and **P < 0.01 *vs.* normoxic control.

Oxidative DNA damage was determined by staining with anti-8-hydroxy-2’-deoxyguanosine (8-OHdG) antibody [18]. 8-OHdG staining was localized in nuclei, and the 8-OHdG-positive cells were stained throughout the cortex penumbra in normoxic group. In the NBO groups, the number of 8-OHdG-positive cells significantly decreased compared with normoxic group (Figure 4). Moreover, longer NBO duration was associated with fewer 8-OHdG-positive cells in cortex penumbra. In the contralateral side of normoxia or NBO groups, positive staining was rarely observed. The 8-OHdG-positive cells were also observed throughout the subcortex (striatal) in both normoxia and NBO groups, and there was no significant difference among groups (data not shown). These findings provide another evidence on neuroprotective effect of NBO via decreasing oxidative stress.

### Effect of NBO on DNA fragmentation

**Figure 5:**
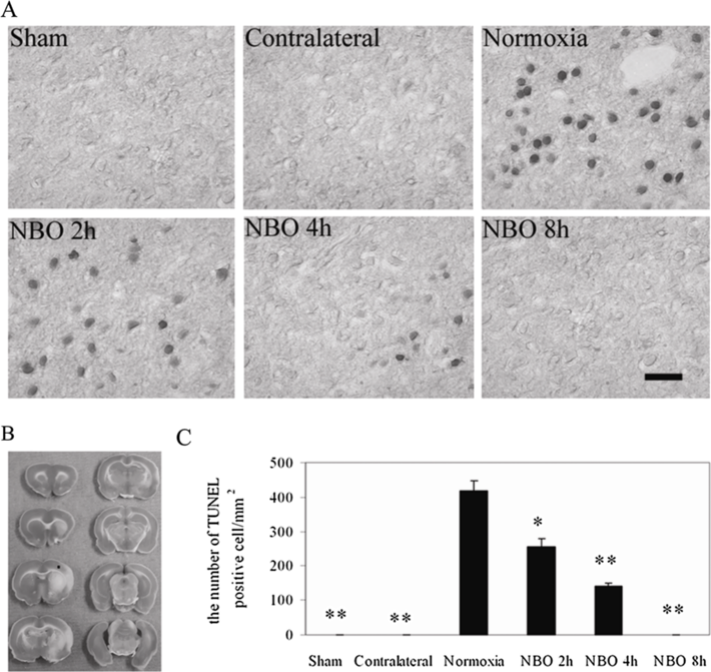
**Effect of NBO on DNA fragmentation in cortex penumbra at 22.5 h reperfusion after 90-min ischemia. A)** Representative photographs showing the effect of NBO on DNA fragmentation by TUNEL in cortex penumbra. Bar = 20 *μ*m. **B)** TTC staining. The black square indicates where the photos were taken from. **C)** Numbers of TUNEL-positive cell in the cortex penumbra. Data is expressed as mean ± SD, *n* = 5 each group. *P < 0.05 and **P < 0.01 *vs.* normoxic control.

To study the impact of NBO on apoptosis in penumbra, we used *in situ* TUNEL staining to evaluate DNA fragmentation. As shown in Figure 5, the staining was localized in nuclei and the TUNEL-positive cells were detected throughout the cortex penumbra in the normoxic group. In the NBO groups, the number of TUNEL-positive cells significantly decreased compared with normoxic group. Moreover, longer NBO duration produced fewer TUNEL-positive cells in cortex penumbra. In the contralateral side of normoxia or NBO groups, positive staining was rarely observed. The TUNEL-positive cells were stained throughout the subcortex (striatal) in both normoxia and NBO groups, and there was no significant difference among groups (data not shown). These results suggest that NBO protects the cortex penumbra against stroke through the reduction of cell apoptosis.

### Effect of NBO on caspase-3 activity

**Figure 6:**
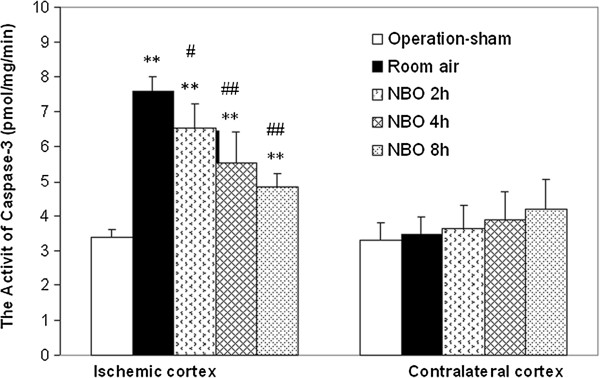
**Effect of NBO on the activity of caspase-3 in cortex penumbral at 22.5 h reperfusion after 90-min ischemia.** Data is expressed as mean ± SD, *n* = 5 in each group. *P < 0.05 and **P < 0.01*vs.* operation-sham; # *P* < 0.05 and *## P* < 0.01 *vs.* normoxic group.

TUNEL staining indicates DNA damage, but its specificity for apoptosis is questionable, so we further detected caspase-3 activity to confirm apoptosis [19]. As show in Figure 6, focal cerebral ischemia induced a substantial increase in caspase-3 activity in the cortex penumbra. NBO significantly attenuated the up-activation of caspase-3 caused by ischemia in a NBO duration-dependent manner, decreasing caspase-3 activity in the cortex penumbra by 14%, 27% and 35%, respectively, with 2 h, 4 h or 8 h duration. There was a little increase in caspase-3 activity in contralateral sides in the NBO groups, but there was no significant difference compared with normoxia control. These results further demonstrate that the neuroprotection of NBO is associated with the reduction of cell apoptosis.

## Discussion

The results presented here demonstrate that 2 h, 4 h and 8 h NBO treatment, started at 30 min post ischemia, could successfully reduce the infarction volume caused by 90 min ischemia and 22.5 h reperfusion. Among 2 h, 4 h and 8 h NBO, 8 h NBO had the greatest benefit in reducing 24-hour infarction volume, attenuating oxidative stress and alleviating apoptosis in ischemic penumbra in acute focal cerebral ischemic rats.

The idea of brain injury due to reperfusion and the theoretical potential for exacerbating damage due to the detrimental tissue effects of oxygen-derived free radicals has been a significant concern, raising doubt about the rationale for oxygen as a therapeutic agent [20]. Recent studies, however, suggest that the benefit of hyperoxia may exceed the risk for enhanced oxidative stress. Flynn and Auer found that high oxygen during reperfusion is beneficial, conflicting with the free radical hypothesis of reperfusion injury [1]. Ulatowski et al. found that deliberate hypoxia during reperfusion provided no benefit [21]. Singhal et al. demonstrated that normobaric hyperoxia treatment during focal cerebral ischemia–reperfusion does not increase oxidative stress, as measured by heme oxygenase-1 induction and protein carbonyl formation in the samples of whole hemisphere [11]. Using HPLC-EC, we reported that NBO during ischemia attenuated the production of 8-OHdG and NBO during reperfusion didn’t increase the production of 8-OHdG in the ischemic penumbra [14]. In this study, using immunohistochemistry, we found there was no significant difference in the 8-OHdG-positive cells in the subcortex (striatal) in both normoxia and NBO groups. Moreover, longer NBO duration produced fewer number of 8-OHdG-positive cells in cortex penumbra (Figure 4). In consistent with the result of 8-OHdG production, NBO treatment markedly attenuated the up-expression of NADPH oxidase in a NBO duration-dependent manner in the ischemic penumbra (Figure 3). Interestingly, Angelos et al. found that hypoxic reperfusion generates an increased ROS burst in ischemic heart [22]. These findings seem to suggest that by attenuating long-lasting oxygen deficiency due to ischemia, longer-lasting NBO treatment might better improve cellular function and suppress the production of free radical. However, how hypoxia and hyperoxia affect the generation of free radicals in vivo in cerebrums tissue need further study.

Abundant evidence suggests that apoptosis plays an important role in neuronal cell death in the penumbra and is involved in the recruitment of potentially salvageable penumbral tissue into infarction [23]. Therefore, we investigated the impact of NBO on apoptosis in penumbra by evaluating DNA fragmentation and caspase-3 activity. In accord with the impact of NBO on oxidative stress, NBO treatment duration-dependently attenuated the induction of apoptosis that was associated with reduced DNA fragment and caspase-3 activity. There are similar reports about the impact of oxygen therapy on ischemia-induced apoptosis. Yin et al. reported that hyperbaric oxygen therapy (2.5 atmospheres, 2 h) at 6 h after reperfusion prevented apoptotic death in ischemic cortex in a rat model of focal cerebral ischemia [24]. Liu et al. demonstrated that normobaric hyperoxia treatment significantly decreased caspase-8 cleavage in the penumbra by 31% and 25% during MCAO and reperfusion, respectively [14].

We started NBO therapy from 30 min after ischemia onset and the treatment lasted continuously during reperfusion to maximize its potential benefit, as continuous intra-ischemic and reperfusion NBO therapy offers the greatest benefit [1]. Since better outcome of NBO treatment is associated with earlier start of the treatment, we selected for this study the time point of 30 min post ischemia, within which NBO could be instituted by the patients at home, or by emergency medical technician upon arrival or on ambulance to the hospital. Later start of the treatment may still offer beneficial effects although at a reduced level. Therefore, this study may have important ramifications for eventual human treatment.

The positive outcomes with extended NBO treatment in this study can be attributed to several factors. First, NBO administrated timely at 30 min after ischemia onset. The therapeutic time window for NBO in rodents is short (approximately 60 minutes); initiating treatment at earlier time points enhances the degree of neuroprotection [11]. Early hyperoxia administration may stop the process of ischemic infarct growth by rapidly restoring oxygen and energy to the ischemic but still viable tissue [25]. Second, it’s a 90 minutes transient focal ischemia. The efficacy of hyperoxia diminishes with longer period of ischemia [26] and no beneficial was shown in permanent focal ischemia from NBO treatment [27]. Beynon et al. failed to observe neuroprotection of 5 h NBO treatment in 150 min MCAO rats, and the longer ischemia duration might be one reason [28]. Longer-duration or permanent ischemia is a severe insult that may overwhelm any beneficial effect of hyperoxia therapy. Furthermore, administration of hyperoxia in severely ischemic tissue could result in an augmented susceptibility of mitochondria to oxidative stress, which has been shown to exacerbate cerebral infarct after permanent focal cerebral ischemia in mice [29].

In conclusion, the present study demonstrates that under our experimental conditions, longer duration of NBO therapy is more effective in reducing infarction volume, oxidative damage and alleviating apoptosis in ischemic penumbra than shorter durations. The findings from this study provide an important insight in designing optimal NBO treatment regimen for stroke patients.

## Abbreviations

NBO: Normobaric hyperoxia therapy; MCAO: Middle cerebral artery occlusion; ECA: External carotid artery; LDF: Lasr Doppler flowmetry; TTC: 2,3,5-triphenyltetrazolium chloride; TUNEL: Terminal deoxynucleotidyl transferase-mediated uridine 5’-triphosphate-biotin nick end labeling; 8-OHdG: 8-hydroxy-deoxyguanosine; TdT: Terminal deoxynucleotidyl transferase.

## Competing interests

The authors declare that they have no competing interests.

## Authors’ contributions

ZY participated in the design of the study and carried out all the animal experiments. RP participated in data processing and manuscript writing. WL helped with the experimental design and data analysis and interpretation. KJL participated in the overall design of the study and manuscript writing, and obtained the funding for the project. All authors have read and approved the final manuscript.

## References

[B1] Flynn EP Auer RN Eubaric hyperoxemia and experimental cerebral infarction Ann Neurol 2002 52 5 566 572 12402253 10.1002/ana.10322

[B2] Qi Z Liu W Luo Y Ji X Liu KJ Normobaric hyperoxia-based neuroprotective therapies in ischemic stroke Med Gas Res 2013 3 1 2 23298701 10.1186/2045-9912-3-2 PMC3552719

[B3] Henninger N Bouley J Nelligan JM Sicard KM Fisher M Normobaric hyperoxia delays perfusion/diffusion mismatch evolution, reduces infarct volume, and differentially affects neuronal cell death pathways after suture middle cerebral artery occlusion in rats J Cereb Blood Flow Metab 2007 27 9 1632 1642 17311078 10.1038/sj.jcbfm.9600463

[B4] Liu W Chen Q Liu J Liu KJ Normobaric hyperoxia protects the blood brain barrier through inhibiting Nox2 containing NADPH oxidase in ischemic stroke Med Gas Res 2011 1 1 22 22146586 10.1186/2045-9912-1-22 PMC3231818

[B5] Esposito E Mandeville ET Hayakawa K Singhal AB Lo EH Effects of normobaric oxygen on the progression of focal cerebral ischemia in rats Exp Neurol 2013 249 33 38 23958492 10.1016/j.expneurol.2013.08.005 PMC3805377

[B6] Singhal AB Ratai E Benner T Vangel M Lee V Koroshetz WJ Schaefer PW Sorensen AG Gonzalez RG Magnetic resonance spectroscopy study of oxygen therapy in ischemic stroke Stroke 2007 38 10 2851 2854 17761914 10.1161/STROKEAHA.107.487280

[B7] Chiu EH Liu CS Tan TY Chang KC Venturi mask adjuvant oxygen therapy in severe acute ischemic stroke Arch Neurol 2006 63 5 741 744 16682544 10.1001/archneur.63.5.741

[B8] Padma MV Bhasin A Bhatia R Garg A Singh MB Tripathi M Prasad K Normobaric oxygen therapy in acute ischemic stroke: a pilot study in Indian patients Ann Indian Acad Neurol 2010 13 4 284 288 21264137 10.4103/0972-2327.74203 PMC3021932

[B9] Henninger N Bratane BT Bastan B Bouley J Fisher M Normobaric hyperoxia and delayed tPA treatment in a rat embolic stroke model J Cereb Blood Flow Metab 2009 29 1 119 129 18766195 10.1038/jcbfm.2008.104

[B10] Singhal AB A review of oxygen therapy in ischemic stroke Neurol Res 2007 29 2 173 183 17439702 10.1179/016164107X181815

[B11] Singhal AB Dijkhuizen RM Rosen BR Lo EH Normobaric hyperoxia reduces MRI diffusion abnormalities and infarct size in experimental stroke Neurology 2002 58 6 945 952 11914413 10.1212/wnl.58.6.945

[B12] Liu S Connor J Peterson S Shuttleworth CW Liu KJ Direct visualization of trapped erythrocytes in rat brain after focal ischemia and reperfusion J Cereb Blood Flow Metab 2002 22 10 1222 1230 12368661 10.1097/01.wcb.0000037998.34930.83

[B13] Reffelmann T Kloner RA Microvascular alterations after temporary coronary artery occlusion: the no-reflow phenomenon J Cardiovasc Pharmacol Ther 2004 9 3 163 172 15378136 10.1177/107424840400900303

[B14] Liu S Liu W Ding W Miyake M Rosenberg GA Liu KJ Electron paramagnetic resonance-guided normobaric hyperoxia treatment protects the brain by maintaining penumbral oxygenation in a rat model of transient focal cerebral ischemia J Cereb Blood Flow Metab 2006 26 10 1274 1284 16421507 10.1038/sj.jcbfm.9600277

[B15] Liu S Shi H Liu W Furuichi T Timmins GS Liu KJ Interstitial pO2 in ischemic penumbra and core are differentially affected following transient focal cerebral ischemia in rats J Cereb Blood Flow Metab 2004 24 3 343 349 15091115 10.1097/01.WCB.0000110047.43905.01

[B16] Komine-Kobayashi M Chou N Mochizuki H Nakao A Mizuno Y Urabe T Dual role of Fcgamma receptor in transient focal cerebral ischemia in mice Stroke 2004 35 4 958 963 14988576 10.1161/01.STR.0000120321.30916.8E

[B17] Walder CE Green SP Darbonne WC Mathias J Rae J Dinauer MC Curnutte JT Thomas GR Ischemic stroke injury is reduced in mice lacking a functional NADPH oxidase Stroke 1997 28 11 2252 2258 9368573 10.1161/01.str.28.11.2252

[B18] Zhang N Komine-Kobayashi M Tanaka R Liu M Mizuno Y Urabe T Edaravone reduces early accumulation of oxidative products and sequential inflammatory responses after transient focal ischemia in mice brain Stroke 2005 36 10 2220 2225 16166574 10.1161/01.STR.0000182241.07096.06

[B19] Namura S Zhu J Fink K Endres M Srinivasan A Tomaselli KJ Yuan J Moskowitz MA Activation and cleavage of caspase-3 in apoptosis induced by experimental cerebral ischemia J Neurosci 1998 18 10 3659 3668 9570797 10.1523/JNEUROSCI.18-10-03659.1998 PMC6793169

[B20] Flamm ES Demopoulos HB Seligman ML Poser RG Ransohoff J Free radicals in cerebral ischemia Stroke 1978 9 5 445 447 705824 10.1161/01.str.9.5.445

[B21] Ulatowski JA Kirsch JR Traystman RJ Hypoxic reperfusion after ischemia in swine does not improve acute brain recovery Am J Physiol 1994 267 5 Pt 2 H1880 H1887 7977818 10.1152/ajpheart.1994.267.5.H1880

[B22] Angelos MG Kutala VK Torres CA He G Stoner JD Mohammad M Kuppusamy P Hypoxic reperfusion of the ischemic heart and oxygen radical generation Am J Physiol Heart Circ Physiol 2006 290 1 H341 H347 16126819 10.1152/ajpheart.00223.2005

[B23] Lei B Popp S Capuano-Waters C Cottrell JE Kass IS Lidocaine attenuates apoptosis in the ischemic penumbra and reduces infarct size after transient focal cerebral ischemia in rats Neuroscience 2004 125 3 691 701 15099683 10.1016/j.neuroscience.2004.02.034

[B24] Yin D Zhou C Kusaka I Calvert JW Parent AD Nanda A Zhang JH Inhibition of apoptosis by hyperbaric oxygen in a rat focal cerebral ischemic model J Cereb Blood Flow Metab 2003 23 7 855 864 12843789 10.1097/01.WCB.0000073946.29308.55

[B25] Anderson DC Zhou C Kusaka I Calvert JW Parent AD Nanda A Zhang JH A pilot study of hyperbaric oxygen in the treatment of human stroke Stroke 1991 22 9 1137 1142 1926256 10.1161/01.str.22.9.1137

[B26] Lou M Eschenfelder CC Herdegen T Brecht S Deuschl G Therapeutic window for use of hyperbaric oxygenation in focal transient ischemia in rats Stroke 2004 35 2 578 583 14715976 10.1161/01.STR.0000111599.77426.A0

[B27] Veltkamp R Sun L Herrmann O Wolferts G Hagmann S Siebing DA Marti HH Veltkamp C Schwaninger M Oxygen therapy in permanent brain ischemia: potential and limitations Brain Res 2006 1107 1 185 191 16828721 10.1016/j.brainres.2006.05.108

[B28] Beynon C Sun L Marti HH Heiland S Veltkamp R Delayed hyperbaric oxygenation is more effective than early prolonged normobaric hyperoxia in experimental focal cerebral ischemia Neurosci Lett 2007 425 3 141 145 17850964 10.1016/j.neulet.2007.07.009

[B29] Chan PH Kawase M Murakami K Chen SF Li Y Calagui B Reola L Carlson E Epstein CJ Overexpression of SOD1 in transgenic rats protects vulnerable neurons against ischemic damage after global cerebral ischemia and reperfusion J Neurosci 1998 18 20 8292 8299 9763473 10.1523/JNEUROSCI.18-20-08292.1998 PMC6792858

